# Enhancement of OVA-induced murine lung eosinophilia by co-exposure to contamination levels of LPS in Asian sand dust and heated dust

**DOI:** 10.1186/1710-1492-10-30

**Published:** 2014-06-09

**Authors:** Yahao Ren, Takamichi Ichinose, Miao He, Yuan Song, Yasuhiro Yoshida, Seiichi Yoshida, Masataka Nishikawa, Hirohisa Takano, Guifan Sun, Takayuki Shibamoto

**Affiliations:** 1 Department of Nutritional and Food Hygiene, College of Public Health, China Medical University, Shenyang, China; 2 Department of Health Sciences, Oita University of Nursing and Health Sciences, Oita 870-1201, Japan; 3 Department of Environmental and Occupational Health, College of Public Health, China Medical University, Shenyang, China; 4 Department of Immunology and Parasitology, School of Medicine, University of Occupational and Environmental Health, Japan, Fukuoka 807-8555, Japan; 5 Environmental Chemistry Division, National Institute for Environmental Studies, Ibaraki 305-8506, Japan; 6 Environmental Health Division, Department of Environmental Engineering, Graduate School of Engineering, Kyoto University, Kyoto 615-8530, Japan; 7 Department of Environmental Toxicology, University of California, Davis, CA 95616, USA

**Keywords:** Lipopolysaccharide, Asian sand dust, Ovalbumin, Lung eosinophilia, Cytokine and chemokine, Asthma

## Abstract

**Background:**

A previous study has shown that the aggravation of Asian sand dust (ASD) on ovalbumin (OVA)-induced lung eosinphilia was more severe in lipopolysaccharide (LPS)-rich ASD than in SiO_2_-rich ASD. Therefore, the effects of different LPS contamination levels in ASD on the aggravation of OVA-induced lung eosinophilia were investigated in the present study.

**Methods:**

Before beginning the *in vivo* experiment, we investigated whether the ultra-pure LPS would act only on TLR4 or not using bone marrow-derived macrophages (BMDMs) of wild–type, TLR2-/-, TLR4-/- and MyD88-/- BALB/c mice. ASD collected from the desert was heated to remove toxic organic substances (H-ASD). BALB/c mice were instilled intratracheally with 12 different testing samples prepared with LPS (1 ng and 10 ng), H-ASD, and OVA in a normal saline solution. The lung pathology, cytological profiles in the bronchoalveolar lavage fluid (BALF), the levels of inflammatory cytokines/chemokines in BALF and OVA-specific immunoglobulin in serum were investigated.

**Results:**

The LPS exhibited no response to the production of TNF-α and IL-6 in BMDMs from TLR4-/-, but did from TLR2-/-. H-ASD aggravated the LPS-induced neutrophilic lung inflammation. In the presence of OVA, LPS increased the level of eosinophils slightly and induced trace levels of Th2 cytokines IL-5 and IL-13 at the levels of 1 ng and 10 ng. In the presence of OVA and H-ASD, LPS induced severe eosinophil infiltration and proliferation of goblet cells in the airways as well as remarkable increases in Th2 cytokines IL-5 and IL-13 in BALF. The mixture containing LPS (1 ng) showed adjuvant activity on OVA-specific IgE and IgG1 production.

**Conclusions:**

The results suggest that H-ASD with naturally-occurring levels of LPS enhances OVA-induced lung eosinophilia via increases in Th2-mediated cytokines and antigen-specific immunoglobulin. These results indicate that LPS is a strong candidate for being a major aggravating substance in ASD.

## Background

Asian sand dust (ASD) is observed most frequently in the spring. When a large scale sandstorm occurs in Northern China and Mongolia, ASD aerosol spreads over large areas, including East China, the Korean Peninsula and Japan as well as crossing the North Pacific to the United States [1-3]. In recent years, ASD has received more and more attention as the adverse effects on human health become known. Epidemiological studies suggested that ASD events may be associated with increased death rates due to respiratory and circulatory diseases [4,5]. Some reports also suggest that ASD events are associated with increased respiratory symptoms in both adults and children with asthma [6,7].

Our previous studies have shown that ASD had aggravating effects on ovalbumin (OVA) - induced lung eosinophilia, whereas ASD heated at 360°C to exclude organic substances and chemicals (H-ASD) caused fewer effects [8,9]. On the basis of these results, we speculate that the organic substances and chemicals adhering to ASD may contribute to the aggravation of lung eosinophila. Microbial and by-product materials derived from air-pollutants, including polycyclic aromatic hydrocarbons (PAHs), sulfates $\left({\text{SO}}_{4}^{\;2-}\right)$ and nitrates $\left({\text{NO}}_{3}^{\;-}\right)$, adhere onto ASD during long-range transportation of the dust [10-12]. ASD was contaminated with trace levels of LPS, which is a cell wall component of gram-negative bacteria. Epidemiological studies suggest that endotoxin levels in samples from children’s mattress were inversely related to the occurrence of atopic asthma [13]. On the other hand, some other studies have shown that endotoxin exposure is positively associated with an increased risk of asthma and asthma severity in both adults and children [14,15]. The results of animal studies are also somewhat contradictory. Interestingly, some studies suggest that LPS at a low dose facilitates a Th2 response to allergens, while LPS at a high dose favors Th1 inflammation [16,17]. Our recent study has shown that the aggravation of murine lung eosinophilia was more severe in LPS-rich ASD than in SiO_2_-rich ASD [18]. Therefore, experimental study as to whether the levels of LPS contamination in ASD are significantly related to the degree of aggravation of the lung eosinophilia is in order.

In the present study, the exacerbating effects of low levels of LPS and/or H-ASD on OVA-induced lung eosinophilia were investigated using BALB/c mice.

## Materials and methods

### Animals

Specific pathogen-free male BALB/c mice (6 weeks of age) were obtained from Charles River Japan, Inc. (Kanagawa, Japan). The body weight of the mice and the presence of infection were checked for 1 week. The mice used were 7 weeks of age. CE-2 commercial diet (CLEA Japan, Tokyo, Japan) and water were given *ad libitum*. The mice were housed in plastic cages lined with soft wood chips. The cages were placed in a conventional room, which was air conditioned at 23°C with a light/dark (12 h/12 h) cycle, and humidity ranging from 55 to 70%. The study adhered to the US National Institutes of Health guidelines for the use of experimental animals. The animal care method was also approved by the animal care and use committee at Oita University of Nursing and Health Sciences in Oita, Japan.

### Preparation of particles and LPS

The Asian dust used as the standard base for the samples in this study was collected from surface soils in the Gobi desert and purified for use in the present study. The size distribution peak was observed at 3.9 μm. The chemical elements in ASD were as reported previously: 51.6% SiO_2_, 14.3% Al_2_O_3_, 5.5% Fe_2_O_3_, 1.3% Na_2_O, 9.6% CaCO_3_, 0.6% CaO, 2.5% MgO, 0.7% TiO_2_ and 2.6% K_2_O. And, as in the previous study, 11.3% of total oxides were lost at ig [19]. A portion of the standard ASD was heated at 360°C for 30 min in an electric heater to exclude toxic materials (sulfate, nitrate, microorganism, etc.). These samples are termed H-ASD in the present study. Ultra pure LPS was purchased from InvivoGen (San Diego, CA, USA).

### Study protocol

One hundred and sixty eight BALB/c mice were divided into twelve groups (n = 14 per group) and each group was treated with a specific testing sample. The 12 testing samples (0.1 mL each of 0.9% NaCl normal saline solution) prepared for the present study were control (containing normal saline alone); H-ASD (0.1 mg H-ASD alone); LPS 1 (1 ng LPS alone); LPS 10 (10 ng LPS alone); H-ASD + LPS 1 (0.1 mg H-ASD and 1 ng LPS); H-ASD + LPS 10 (0.1 mg H-ASD and 10 ng LPS); OVA (2 μg OVA alone); OVA + H-ASD (2 μg OVA and 0.1 mg H-ASD); OVA + LPS 1 (2 μg OVA and 1 ng LPS); OVA + LPS 10 (2 μg OVA and 10 ng LPS); OVA + H-ASD + LPS 1 (2 μg OVA, 0.1 mg H-ASD, and 1 ng LPS); and OVA + H-ASD + LPS 10 (2 μg OVA, 0.1 mg H-ASD, and 10 ng LPS). The mice were intratracheally challenged with a mixed or individual solution of OVA, H-ASD and LPS 4 times at 2-week intervals. The control group was instilled intratracheally with 0.1 ml normal saline.

### Bronchoalveolar lavage fluid (BALF)

Eight out of 14 mice were used for an examination of the free cell contents in BALF. BALF and cell counts were conducted using a previously reported method [18,20]. Briefly, the lungs were lavaged with two injections of 0.8 ml of sterile saline at 37°C. After the fluids from the two lavages were combined and cooled to 4°C, the resultant solution was centrifuged at 1500 rpm for 10 min. The protein levels of cytokines and chemokines in the BALF were measured. The total cell count of the fresh fluid specimen was determined by a hemocytometer. Differential cell counts were assessed on cytological preparations. Slides were prepared using a Cytospin (Sakura Co., Ltd, Tokyo, Japan) and stained with Diff-Quik (International Reagents Co., Kobe, Japan) to identify the eosinophils with red granules. A total of 300 cells were counted under a microscope. The BALF supernatants were stored at -80°C to await analysis for cytokines and chemokines.

### Pathological evaluation

The remaining 6 mice in each group were used for pathological examination. The lungs were fixed by 10% neutral phosphate-buffered formalin. After separation of the lobes, 2-mm-thick blocks were taken for paraffin embedding. Embedded blocks were sectioned at a thickness of 3 μm, and then stained with hematoxylin and eosin (H & E) to evaluate the degree of infiltration of eosinophils and lymphocytes in the airway from proximal to distal. The sections were stained with periodic acid-Schiff (PAS) to evaluate the degree of proliferation of goblet cells in the bronchial epithelium. A pathological analysis of inflammatory cells and epithelial cells in the airway was performed using a Nikon ECLIPSE light microscope (Nikon Co., Tokyo, Japan). The degree of infiltration of eosinophils and lymphocytes in the airway or proliferation of goblet cells in the bronchial epithelium was graded in a blinded fashion: 0, not present; 1, slight; 2, mild; 3, moderate; 4, moderate to marked; 5, marked. ‘Slight’ was defined as less than 20% of the airway with eosinophilic inflammatory reaction or with goblet cells stained with PAS; ‘mild’ as 21 - 40%; ‘moderate’ as 41 - 60%; ‘moderate to marked’ as 61 - 80%; and marked as more than 80% of the airway [18,20].

### Quantitation of cytokines and chemokines in BALF

The cytokine and chemokine protein levels were determined by enzyme-linked immunosorbent assays (ELISA). IL-5 and IL-12 were measured using an ELISA kit from Endogen, Inc. (Cambridge, MA, USA). MCP-3 was measured using an ELISA kit from Bender MedSystems Inc. (Burlingame, CA, USA). IL-1β, IL-4, IL-6, IL-13, IL-17A, IFN-γ, KC, TNF-α, TGF-β, Eotaxin, MCP-1, MIP-1α, RANTES were measured using an ELISA kit from R&D Systems Inc. (Minneapolis, MN, USA).

### OVA-specific IgE and IgG1 antibodies

OVA-specific immunoglobulin E (IgE) and IgG1 antibodies were measured using the Mouse OVA-IgE ELISA kit and Mouse OVA-IgG1 ELISA kit (Shibayagi Co., Shibukawa, Japan). According to the manufacturer’s protocol, 1U of the anti-OVA IgE is defined as 1.3 ng of the antibody; and 1U of the anti-OVA IgG1 is defined as 160 ng of the antibody. The absorption of 450 nm (sub-wave length, 620 nm) for OVA-specific IgE and IgG1 antibody was measured by a microplate reader (Spectrafluor, Tecan, Salzburg, Austria).

### Isolation and culture of murine bone marrow-derived macrophages (BMDMs)

Wild–type, TLR2-/-, TLR4-/- and MyD88-/- mice (on BALB/c background) were purchased from Oriental Bioservice, Inc (Kyoto, Japan). Femurs were removed at 8 weeks of age, the soft tissue removed, and flushed with Hanks to recover the bone marrow. BMDMs were cultured on plastic dishes in RPMI (Nissui, Tokyo, Japan) containing penicillin/streptomycin (Gibco, New York, NY) and 10% inactivated fetal bovine serum (Thermo Scientific, South Logan, UT). 10 ng/ml of GM-CSF (PeproTech EC, London, UK) was added to the culture on days 0 and 3. After 6 days in culture, the adherent BMDMs were collected by adding 0.02% 5 ml EDTA and then scraping the loose cells off. Then BMDMs were re-plated at a concentration of 3 × 10^5^ cells/ml into 24-well plates at 1 ml/well. BMDMs were incubated with PBS or LPS (final 1 μg/ml) for 12 h. Cytokines secreted into the culture medium by BMDMs were measured by ELISA.

### Assay for cytokines and chemokines in cell culture medium

The protein levels of cytokines and chemokines in the nutrient medium were determined by ELISA. IL-6, MCP-1, MIP-1α, and TNF-α were measured using ELISA kits from R&D Systems Inc.

### Statistical analysis

Statistical analysis on the pathological evaluation in the airway, cytokines, and chemokine proteins in BALF were conducted using the Tukey Test for Pairwise Comparisons (KyPlot Ver 5.0, Kyens Lab Inc, Tokyo, Japan). Differences among groups were determined as statistically significant at a level of p < 0.05.

## Results

### Cytokines and chemokines in cell culture medium from mice treated by LPS

**Figure 1:**
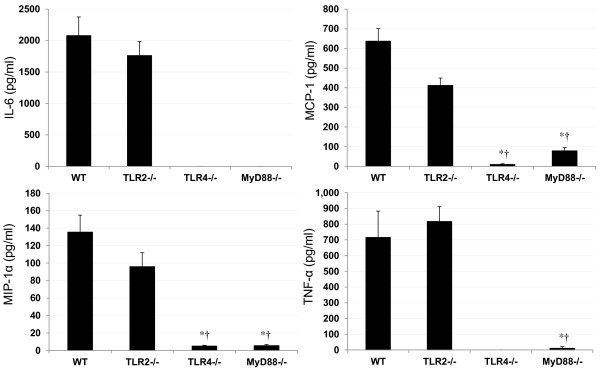
**Cytokine and chemokine production in LPS-stimulated BMDMs of KO mice.** BMDMs were incubated with PBS, LPS (final concentration of 1 μg/ml) for 12 h. The cytokine levels secreted into the culture medium from BMDMs were measured by ELISA. All values were expressed as mean ± SE (n = 6). *p < 0.001 vs. WT; ^†^p < 0.001 vs. TLR2-/-.

Figure 1 shows the amounts of cytokine and chemokine produced in LPS-stimulated BMDMs of KO mice. Relatively high levels of IL-6, MCP-1, MIP-1α and TNF-α were found in WT cells, ranging from 136 ± 19.4 pg/ml (MIP-1α) to 2,080 ± 297 pg/ml (IL-6), and TLR2-/- cells, ranging from 96.0 ± 16.0 pg/ml (MIP-1α) to 1,763 ± 223 pg/ml (IL-6). Trace levels of MIP-1α (5.57 ± 1.20 pg/ml) and TNF-α (10.4 ± 10.4 pg/ml) were observed in MyD88-/- cells, and they reduced significantly compared with WT cells. On the other hand, IL-6 and TNF-α were not detected in TLR4-/- cells. IL-6 was also not detected in MyD88-/- cells.

### The cell numbers in BALF from mice treated by OVA, H-ASD and LPS

**Figure 2:**
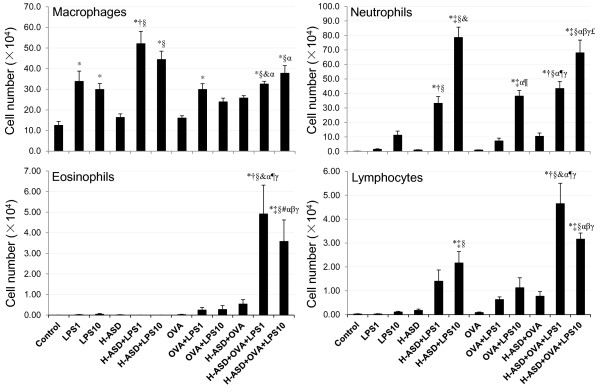
**Cellular profile in bronchoalveolar lavage fluids (BALF).** All values were expressed as mean ± SE. *p < 0.05 vs. control; ^†^p < 0.05 vs. LPS 1; ^‡^p < 0.05 vs. LPS 10; ^§^p < 0.05 vs. H-ASD; ^&^p < 0.05 vs. H-ASD + LPS 1; ^#^p < 0.05 vs. H-ASD + LPS 10; ^α^p < 0.05 vs. OVA; ^¶^p < 0.05 vs. OVA + LPS 1; ^β^p < 0.05 vs. OVA + LPS 10; ^γ^p < 0.05 vs. H-ASD + OVA; ^£^p < 0.05 vs. H-ASD + OVA + LPS 1.

Figure 2 shows the cellular profiles in BALF. The total cell numbers ranged from (12.8 ± 1.92) × 10^4^ (control) to (113 ± 9.33) × 10^4^ (H-ASD + OVA + LPS 10). The cell numbers in the control samples ranged from 0 (eosinophils) to (12.6 ± 1.89) × 10^4^ (macrophages). LPS 1 and LPS 10 increased the number of macrophages by 168% and 137%, respectively. The addition of H-ASD to LPS 1 and LPS 10 increased macrophages, netrophils and lymphocytes significantly, whereas no change was observed in eosinophils. OVA alone did not increase any cell numbers but the addition of LPS 1 and LPS 10 increased all cell numbers in BALF samples slightly. When LPS 1 + H-ASD and LPS 10 + H-ASD were added to OVA, a significant increase of numbers of all cells was observed. The increase was dose dependent in macrophages and neutrophils but not in eosinophils and lymphocytes.

### Cellular changes in the airways of mice treated by OVA, H-ASD and LPS

**Figure 3:**
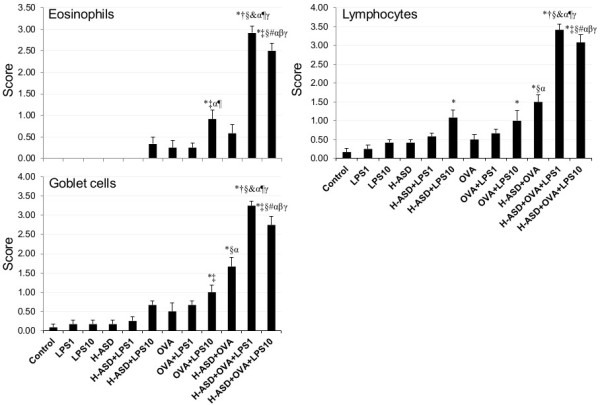
**Evaluation of cellular changes in the murine airway.** The degree of cellular changes in the airway was estimated as: (0) none; (1) slight; (2) mild; (3) moderate; (4) moderate to marked; (5) marked. All values were expressed as mean ± SE (n = 6). Statistical analyses were conducted using Tukey for Pairwise Comparisons. *p < 0.05 vs. control; ^†^p < 0.05 vs. LPS 1; ^‡^p < 0.05 vs. LPS 10; ^§^p < 0.05 vs. H-ASD; ^&^p < 0.05 vs. H-ASD + LPS 1; ^#^p < 0.05 vs. H-ASD + LPS 10; ^α^p < 0.05 vs. OVA; ^¶^p < 0.05 vs. OVA + LPS 1; ^β^p < 0.05 vs. OVA + LPS 10; ^γ^p < 0.05 vs. H-ASD + OVA.

**Figure 4:**
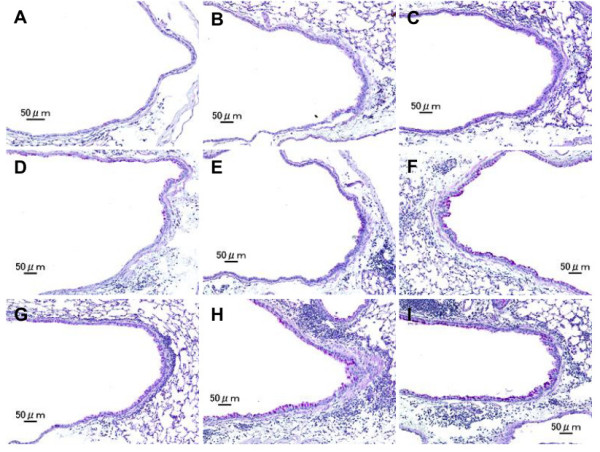
**Effects of test samples on pathological changes in the lungs.** No pathologic alterations were seen in the lungs of the control **(A)**. Slight bronchitis with slight proliferation of goblet cells that have mucus stained pink with PAS was seen in airway epithelium and slight infiltration of inflammatory cells in the submucosa of airways exposed to H-ASD + LPS 1 **(B)**. Bronchitis with slight proliferation of goblet cells and moderate infiltration of inflammatory cells such as neutrophils and lymphocytes were seen in the submucosa of airways exposed to H-ASD + LPS 10 **(C)**. Very slight proliferation of goblet cells in the airway epithelium and very slight infiltration of inflammatory cells such as lymphocytes were seen in the submucosa of airways exposed to OVA alone **(D)**. The pathological alteration in the airway epithelium and the submucosa of airways exposed to OVA + LPS 1 **(E)** were almost the same. The pathological changes exposed to OVA + LPS 10 **(F)** were somewhat stronger than those of OVA + LPS 1. Slight goblet cell proliferation and mild infiltration of inflammatory cells were found in the submucosa of airways exposed to H-ASD + OVA **(G)**. Moderate goblet cell proliferation, accumulation of numerous inflammatory cells in the submucosa of airway, and fibrous thickening of the subepithelial layer were seen in the airways exposed to H-ASD + OVA + LPS 1 **(H)** and moderate goblet cell proliferation, severe infiltration of inflammatory cells in the submucosa of airways exposed to H-ASD + OVA + LPS 10 **(I)**. **(A–I)** PAS stain; bar = 50 μm.

**Figure 5:**
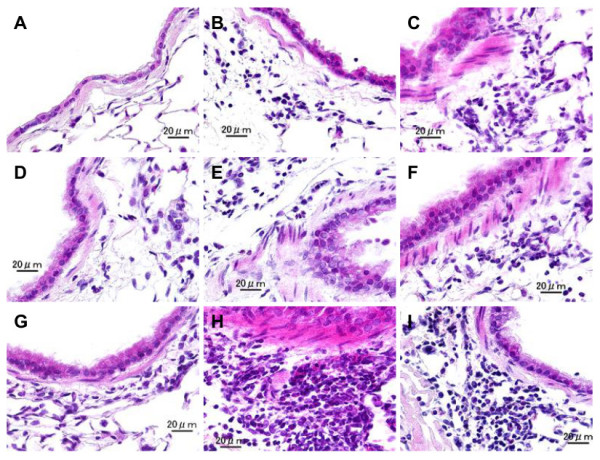
**Effects of test samples on infiltration of inflammatory cells in the airway.** No pathologic changes in the lungs of the control **(A)**. Mild infiltration of neutrophils and lymphocytes in the submucosa of airways exposed to H-ASD + LPS 1 **(B)**. Moderate infiltration of neutrophils and lymphocytes in the submucosa of airways exposed to H-ASD + LPS 10 **(C)**. Slight eosinophils, neutrophils and lymphocytes in the submucosa of airways exposed to OVA alone **(D)**. Mild to moderate infiltration of the inflammatory cells in the submucosa of airways exposed to OVA + LPS 1 **(E)** and OVA + LPS 10 **(F)**. Slight infiltration of inflammatory cells in the submucosa of airways exposed to H-ASD + OVA **(G)**. Severe accumulation of eosinophils, neutrophils and lymphocytes in the submucosa of airways exposed to H-ASD + OVA + LPS 1 **(H)**. Moderate to marked infiltration of eosinophils, neutrophils and lymphocytes in the submucosa of airways exposed to H-ASD + OVA + LPS 1 **(I) (A–I)** HE stain; bar = 20 μm.

Figure 3 shows the cellular changes caused by H-ASD and LPS in the murine airway. The pathological score ranged from 0 (eosinophils) to 3.42 ± 0.15 (lymphocytes). When the control, LPS 1, LPS 10, H-ASD and H-ASD + LPS 1 samples were exposed, a moderate increase in lymphocytes, ranging from 0.25 ± 0.11 (LPS 1) to 0.58 ± 0.08 (H-ASD + LPS 1), was observed along with a slight increase of goblet cells ranging from 0.17 ± 0.11 (LPS 1, LPS 10, H-ASD) to 0.25 ± 0.11 (H-ASD + LPS 1). Eosinopils were not detected. When H-ASD + LPS 10, OVA, OVA + LPS 1, OVA + LPS 10 and H-ASD + OVA were exposed, moderate increases of all cells ranged from 0.25 ± 0.11 (eosinophils) to 1.67 ± 0.25 (goblet cells) occurred. The samples exposed to H-ASD + OVA + LPS 1 and H-ASD + OVA + LPS 10 showed a significant increase in goblet cells (3.25 ± 0.11 and 2.75 ± 021, respectively) eosinophils (2.92 ± 0.15 and 2.50 ± 0.18, respectively) and lymphocytes (3.42 ± 0.15 and 3.08 ± 0.20, respectively) in the submucosa of the airway. It is interesting that the pathological change was greater with low dose (1 ng) addition of LPS than by with 10 ng LPS.Figures 4 and 5 illustrate the effects of LPS on pathological changes in the lungs. No pathological alterations were found in the lungs of the control group. LPS 1 and LPS 10 caused slight infiltration of neutrophils in the submucosa of the airway, while H-ASD + LPS 1 and H-ASD + LPS 10 caused slight proliferation of goblet cells in the airway epithelium (Figure 4B and C) and slight to moderate infiltration of neutrophils in the airway (Figure 5B and C). OVA alone caused a slight proliferation of goblet cells in the airway epithelium (Figure 4D) as well as eosinophils, neutrophils and lymphocytes in the submucosa of airway (Figure 5D).The pathological alteration in the airway epithelium and the submucosa of airways exposed to OVA alone and OVA + LPS 1 were almost the same (Figure 4D, F; Figure 5D, F). However, the pathological changes in samples exposed to OVA + LPS 10 were somewhat higher than those exposed to OVA + LPS 1 (Figure 4E and F). H-ASD + OVA also caused slight goblet cell proliferation and slight infiltration of eosinophils and lymphocytes in the submucosa of airway. However, H-ASD + OVA + LPS 1 and H-ASD + OVA + LPS 10 caused moderate goblet cell proliferation in the airway epithelium (Figure 4H and I) and moderate to marked accumulation of eosinophils, neutrophils and lymphocytes in the submucosa of airways (Figure 5H and I). These pathological alterations in the H-ASD + OVA + LPS 1 group were more severe than in the H-ASD + OVA + LPS 10 group.

### Protein levels of cytokines and chemokines in BALF from mice treated by OVA, H-ASD and LPS

**Figure 6:**
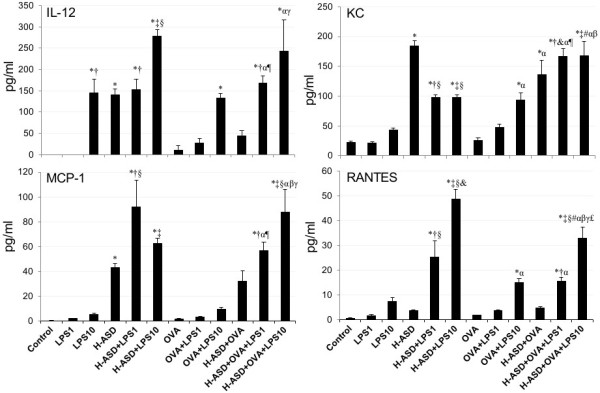
**Expressions of MCP-1, KC, IL-12 and RANTES in BALF.** All values were expressed as mean ± SE (n = 8). *p < 0.05 vs. control; ^†^p < 0.05 vs. LPS 1; ^‡^p < 0.05 vs. LPS 10; ^§^p < 0.05 vs. H-ASD; ^&^p < 0.05 vs. H-ASD + LPS 1; ^#^p < 0.05 vs. H-ASD + LPS 10; ^α^p < 0.05 vs. OVA; ^¶^p < 0.05 vs. OVA + LPS 1; ^β^p < 0.05 vs. OVA + LPS 10; ^γ^p < 0.05 vs. H-ASD + OVA; ^£^p < 0.05 vs. H-ASD + OVA + LPS 1.

**Figure 7:**
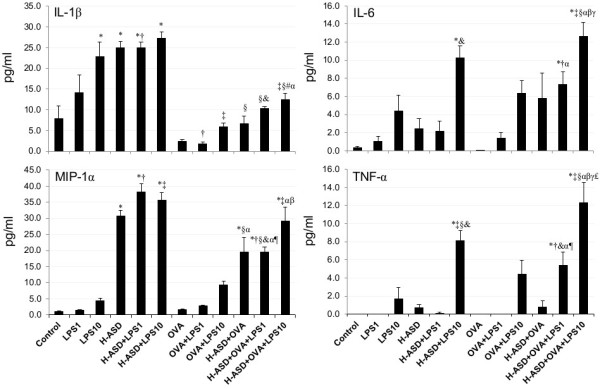
**Expressions of TNF-α, IL-6, IL-1β and MIP-1α in BALF.** All values were expressed as mean ± SE (n = 8). *p < 0.05 vs. control; ^†^p < 0.05 vs. LPS 1; ^‡^p < 0.05 vs. LPS 10; ^§^p < 0.05 vs. H-ASD; ^&^p < 0.05 vs. H-ASD + LPS 1; ^#^p < 0.05 vs. H-ASD + LPS 10; ^α^p < 0.05 vs. OVA; ^¶^p < 0.05 vs. OVA + LPS 1; ^β^p < 0.05 vs. OVA + LPS 10; ^γ^p < 0.05 vs. H-ASD + OVA; ^£^p < 0.05 vs. H-ASD + OVA + LPS 1.

**Figure 8:**
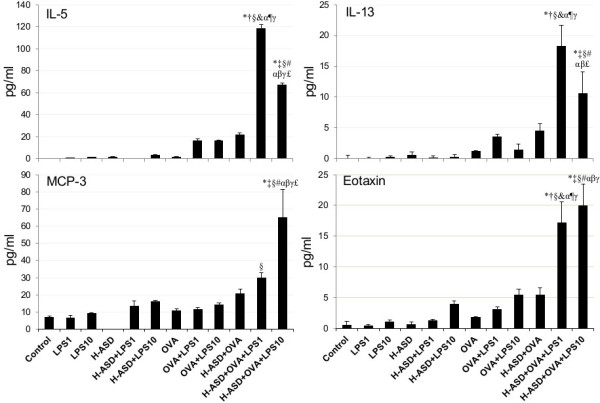
**Expressions of IL-5, IL-13, Eotaxin and MCP-3 in BALF.** All values were expressed as mean ± SE (n = 8). *p < 0.05 vs. control; ^†^p < 0.05 vs. LPS 1; ^‡^p < 0.05 vs. LPS 10; ^§^p < 0.05 vs. H-ASD; ^&^p < 0.05 vs. H-ASD + LPS 1; ^#^p < 0.05 vs. H-ASD + LPS 10; ^α^p < 0.05 vs. OVA; ^¶^p < 0.05 vs. OVA + LPS 1; ^β^p < 0.05 vs. OVA + LPS 10; ^γ^p < 0.05 vs. H-ASD + OVA; ^£^p < 0.05 vs. H-ASD + OVA + LPS 1.

Figure 6 shows the levels of IL-12, KC, MCP-1 and RANTES in BALF. The protein levels in the control ranged from not detected (IL-12) to 22.8 ± 1.27 pg/ml (KC). LPS 1 alone did not significantly increase the proteins examined, whereas LPS 10 increased them appreciably, to levels ranging from 5.14 ± 1.28 pg/ml (MCP-1) to 145 ± 32.5 pg/ml (IL-12). H-ASD increased IL-12, KC, and MCP-1. H-ASD + LPS increased all proteins. In particular, the addition of LPS 10 to H-ASD increased IL-12 by 99% and RANTES by 1179%. The amount of proteins found in the samples treated with OVA alone ranged from 1.39 ± 0.52 pg/ml (MCP-1) to 25.8 ± 4.17 (KC). The addition of LPS to OVA showed a dose-dependent increase in all proteins. When LPS was added to an OVA + H-ASD sample, all proteins also showed a dose-dependent increase.Figure 7 shows the expression of IL-1β, IL-6, MIP-1α and TNF-α. The protein levels in the control samples ranged from not detected (TNF-α) to 7.82 ± 3.17 pg/ml (IL-1β). LPS increased all proteins dose-dependently. H-ASD increased but OVA reduced all proteins. H-ASD alone increased IL-1β significantly, by 218%. Addition of LPS 10 to H-ASD increased IL-6 by 327% but LPS 1 reduced IL-6 slightly (by 11%). The amount of proteins found in samples treated by OVA ranged from 0 (TNF-α) to 2.31 ± 0.51 pg/ml (IL-1β). Addition of LPS 10 to OVA increased IL-1β from 2.31 ± 0.51 pg/ml to 5.92 ± 0.82 pg/ml, whereas addition of LPS 1 to OVA decreased it to 1.79 ± 0.44 pg/ml. Addition of LPS to H-ASD + OVA dose-dependently increased all proteins considerably. The mice treated by H-ASD + OVA + LPS 10 contained the highest levels of all proteins, ranging from 12.3 ± 2.3 pg/ml (TNF-α) to 29.2 ± 4.31 pg/ml (MIP-1α).Figure 8 shows the expression of IL-5, IL-13, eotaxin and MCP-3 in BALF. These proteins are known as allergy associated mediators. The amount in the control group of samples ranged from not detected (IL-13) to 6.76 ± 0.94 pg/ml (MCP-3). Among the proteins in this group, MCP-3 was detected in the highest levels in all samples from treated mice except one set of sample from H-ASD treatment, ranging from not detected (H-ASD) to 65.3 ± 16.3 pg/ml (H-ASD + OVA + LPS 10). When LPS was added to H-ASD, slight, dose-dependent increases of all proteins were observed.

**Figure 9:**
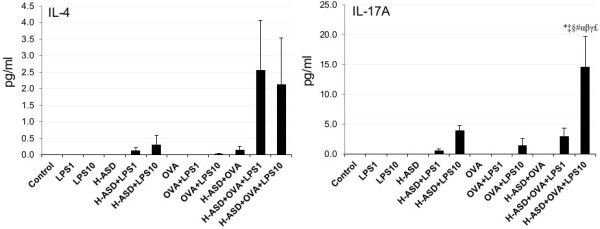
**Expressions of IL-4 and IL-17A in BALF.** All values were expressed as mean ± SE (n = 8). *p < 0.05 vs. control; ^†^p < 0.05 vs. LPS 1; ^‡^p < 0.05 vs. LPS 10; ^§^p < 0.05 vs. H-ASD; ^&^p < 0.05 vs. H-ASD + LPS 1; ^#^p < 0.05 vs. H-ASD + LPS 10; ^α^p < 0.05 vs. OVA; ^β^p < 0.05 vs. OVA + LPS 1; ^γ^p < 0.05 vs. H-ASD + OVA; ^£^p < 0.05 vs. H-ASD + OVA + LPS 1.

A slight increase of all proteins was observed in the samples treated by OVA alone, ranging from 1.14 ± 0.38 pg/ml (IL-13) to 10.8 ± 0.87 pg/ml (MCP-3). Addition of LPS to OVA increased IL-5 significantly but the other samples increased only slightly. Addition of H-ASD to OVA increased IL-5 considerably from 1.68 ± 0.51 pg/ml to 21.5 ± 6.78 pg/ml. The addition of LPS to OVA + H-ASD triggered a remarkable increase in all proteins. However, the increasing levels of IL-5 and IL-13 in the group treated with LPS 1 (448% and 302%, respectively) were higher than those of the group treated with LPS 10 (211% and 133%, respectively).Figure 9 shows the expression of IL-4 and IL-17A. They were not detected in the samples of the control, LPS 1, LPS 10, or H-ASD groups. The amounts of IL-4 ranged from not detected to 2.55 ± 1.52 pg/ml (H-ASD + OVA + LPS 1) and IL-17A ranged from not detected to 14.6 ± 5.14 (H-ASD + OVA + LPS 10). IL-4 and IL-17A were not present in significant amounts in the samples treated with H-ASD + LPS 1, OVA, OVA + LPS 1 and H-ASD + OVA but H-ASD and LPS obviously increased both proteins.

In the present study, TGF-β and IFN-γ were not detected.

### Enhancement of OVA-specific IgE and IgG1 by H-ASD and LPS

**Figure 10:**
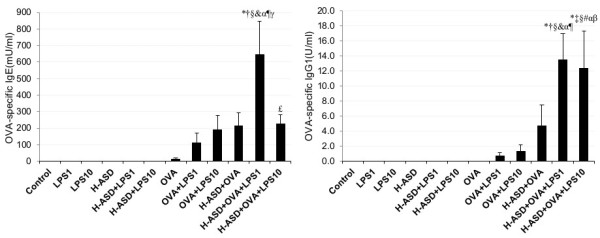
**Effects of test samples on IgE and IgG1 production in serum.** According to the manufacturer’s protocol, 1 U of the anti-OVA IgE is defined as 1.3 ng of the antibody and 1 U of the anti-OVA IgG1 is defined as 160 ng of the antibody. All values were expressed as mean ± SE. *p < 0.05 vs. control; ^†^p < 0.05 vs. LPS 1; ^‡^p < 0.05 vs. LPS 10; ^§^p < 0.05 vs. H-ASD; ^&^p < 0.05 vs. H-ASD + LPS 1; ^#^p < 0.05 vs. H-ASD + LPS 10; ^α^p < 0.05 vs. OVA; ^¶^p < 0.05 vs. OVA + LPS 1; ^β^p < 0.05 vs. OVA + LPS 10; ^γ^p < 0.05 vs. H-ASD + OVA; ^£^p < 0.05 vs. H-ASD + OVA + LPS 1.

Figure 10 shows the effects of testing samples on IgE and IgG1 production in serum. IgE and IgG1 were not detected in the samples of the control, LPS 1, LPS 10, H-ASD, H-ASD + LPS 1 or H-ASD + LPS 10 samples. Trace levels of IgE were observed in OVA but not IgG1. H-ASD + OVA and H-ASD + OVA + LPS 10 increased to similar levels of OVA-specific IgE. H-ASD + OVA + LPS 1 increased the level of the IgE and IgG1 in serum most effectively.

## Discussion

Exposure to LPS is known to be a significant risk factor for increased asthma prevalence and severity [21,22]. Therefore, LPS contaminated ASD may cause aggravation of allergic lung inflammation. Previous reports have demonstrated that co-treatment of TLR2-ligand Pam3Cys and OVA activated an OVA-associated Th2-biased immune response in experimental asthma [23]. Therefore, ultra-pure LPS was used in this study because the LPS made commercially is contaminated with TLR2-ligands. Before beginning the *in vivo* experiment, we investigated whether our ultra-pure LPS would act only on TLR4 or not using BMDMs of WT, TLR2 KO, TLR 4 KO and MyD88 KO mice. As shown in Figure 1, this LPS exhibited no response to the production of cytokines (TNF-α and IL-6) in BMDMs from TLR4 KO, but from TLR2 KO.

Previous studies have reported that LPS inhaled in low levels (0.1 μg) enhanced Th2 responses to inhaled OVA in a mouse model of Th2 pulmonary inflammation, while inhalation of high levels (10 μg, 100 μg) of LPS with an antigen caused a Th1 response [16,17].

In the absence of OVA, 10 ng LPS alone caused a slight increase of neutrophils in BALF, but 1 ng LPS was less effective than10 ng LPS. However in the presence of H-ASD, 10 ng LPS caused a marked increase of neutrophils in BALF along with the proinflammatory mediators IL-12, RANTES, MCP-1, IL-6, and TNF-α. Pathologically, 10 ng LPS caused moderate bronchitis and alveolitis, suggesting that H-ASD aggravates LPS-induced lung inflammation via the increased proinflammatory mediators.In the presence of OVA, LPS at the levels of 1 ng and 10 ng caused a slight increase of eosinophils (Figure 2). LPS at the level of 10 ng caused a moderate increase of neutrophils along with proinflammatory mediators IL-12, KC, RANTES, MCP-1, IL-6 and TNF-α in BALF, but the induction of Th2 cytokines IL-5, IL-13 occurred only at trace levels, suggesting that OVA + LPS 10 does not serve to induce strong Th2 responses.In the presence of H-ASD + OVA, LPS at the levels of 1 ng and 10 ng increased neutrophil and eosinophi numbers. In particular, 10 ng LPS increased more pro-inflammatory cytokines (IL-1β, IL-6, IL-12, IL-17A, TNF-α) and chemokines (RANTES, MIP-1α, KC, MCP-1, Eotaxin, MCP-3) in BALF than 1 ng LPS (Figures 6, 7, 8). However, The pathological changes—eosinophil recruitment in the submucosa of the airway along with proliferation of goblet cells in the bronchial epithelium in the airway— and Th2 cytokines IL-5/IL-13 induced by 1 ng LPS were more remarkable than 10 ng LPS, suggesting that co-exposure to H-ASD and LPS, particularly 1 ng LPS, can lead to Th2 responses to inhaled OVA more than 10 ng LPS can. The Th2 cytokines together with increased eosinophil-relevant chemokines eotaxin and MCP-3 aggravated eosinophilic lung inflammation in the present study.

Overall, H-ASD played an important role in the exacerbation of pathological changes (Figure 3). Eosinophils are reportedly implicated in tissue destruction in allergic asthma [24]. Because toxic eosinophil–derived proteins, such as major basic proteins, cause bronchial mucosal damage in asthmatic airways, they may exacerbate the symptoms of asthma [25]. IL-5 generated from Th2 cells attracts and activates eosinophils, which are implicated in tissue destruction in allergic asthma [26]. IL-13 has been shown to stimulate B cells and subsequently produce antigen specific antibodies [27]. It also promotes mucous secretion and the production of mucous cells, such as goblet cells, in the bronchial epithelium [28]. Therefore, airway injury resulting from co-treatment may be due to exacerbation of eosinophilic airway-inflammation. This allergic airway inflammation recruits not only eosinophils, but also neutrophils. There is a report that IL-17A contributes to neutrophil infiltration in airway inflammation in allergic asthma [29].

Toll like receptors (TLRs) are pattern recognition receptors (PRRs) that play an essential role in animal immunity [30]. TLR4 is well known as a receptor for LPS. Previous *in vivo* findings indicate that low levels of LPS can cause TLR4-dependent Th2 responses to OVA [16,31]. Low levels of LPS activate Th2 responses to OVA through the adaptor protein myeloid differentiation factor 88 (MyD88) [32]. In this animal study, the induction of pro-inflammatory molecules in BALF may be a TLR4-dependent signaling pathway, but not a TLR2 pathway, because there is no contamination of TLR2-ligands in LPS from as a result of the *in vitro* experiment. In the presence of OVA, Th2 responses to 1 ng and 10 ng LPS were defective. Interestingly, in the presence of OVA and H-ASD, the two doses of LPS, especially the 1 ng dose markedly enhanced Th2 responses. This may have been caused by a TLR4-dependent signaling pathway through MyD88. In both the presence and absence of OVA, low dose LPS caused no response in the form of induction of Th1 cytokine IFN-γ, suggesting that low doses of LPS lack the potency for leading to a Th1 response. Interferon γ reportedly inhibits the development of secondary allergic responses in mice [33]. Therefore, we speculate that the lack of IFN-γ skews lung inflammation in a Th2 direction. However, the present study did not provide sufficient evidence to explain the role of H-ASD in enhancement of Th2 responses. Our previous *in vitro* study has shown that H-ASD treatment in the presence of LPS dose-dependently enhanced the production of inflammatory mediators by BMDMs from ICR mice [18]. In this *in vivo* study, co-exposure to H-ASD and LPS enhanced neutrophilic inflammation and the production of inflammatory mediators in BALF. An increase in antigen presenting cells, such as macrophages, caused by H-ASD (Figure 2) and the activation of the TLR4-signaling pathway leading to inflammatory cytokine production by H-ASD may lead to an adaptive immune response related to Th2. Although the pathway leading to Th2 activation is unclear in this study, it is clear that H-ASD has potential to elicit a weak reaction by adding a low dose of LPS. Similar potential of H-ASD has been observed in lung inflammation caused by Gram negative bacteria, *Klebsiella Pneumoniae*[34] and fungi, *Bjerkandera adusta*[35].

On the other hand, in the presence of H-ASD and OVA, low doses of LPS caused the production of OVA-specific IgE and IgG1 in serum. It seems that these increases are being interlocked with activation of Th2 response caused by these mixtures. H-ASD used contains 14.3% Al_2_O_3_. Although aluminum adjuvant is well known, Al_2_O_3_ may not contribute to the adjuvant effect. The adjuvant effect of Al_2_O_3_ particle on their immunoglobulin productions was not detected in our previous study [36]. However, SiO_2_ is a possible adjuvant. Allergen-specific antibodies may contribute to the occurrence of inflammatory cell accumulation after an allergen challenge via IgE-mediated degranulation of mast cells [37]. Antigen-specific IgG1 can cause degranulation via an Fcg RII receptor on the eosinophil’s surface [38]. Therefore, antibodies may play an important role in the aggravation of lung eosinophilia caused by H-ASD + OVA + LPS.

## Conclusions

This study demonstrates that LPS contamination in ASD aggravates allergic lung inflammation in the presence of OVA and H-ASD. The aggravation of the allergic lung inflammation by LPS may be caused through the TLR4-dependent signaling pathway. The results of the current study indicate that the exposure to ASD with LPS may be a significant risk factor for adult and child asthma. The hazardous effects of mineral dust and biogenic agents on human respiratory disease are an increasing public concern. Atmospheric exposure to bacteria, fungi and virus, and silica-carrying particulate matters may influence human respiratory health on a world-wide scale.

## Abbreviations

ASD: Asian sand dust; LPS: Lipopolysaccharide; BALF: Bronchoalveolar lavage fluid; BMDM: Bone marrow-derived macrophages; ELISA: Enzyme-linked immunosorbent assays; EMSA: Electrophoretic mobility shift assay; H-ASD: Heated Asian sand dust; IFN-γ: Interferon-γ; IL: Interleukin; KC: Keratinocyte chemoattractant; MCP-1: Monocyte chemotactic protein-1; MCP-3: Monocyte chemotactic protein-3; MIP-1α: Macrophage inflammatory protein-1α; RANTES: Regulated on activation normal T cell expressed and presumably secreted; TNF-α: Tumor necrosis factor-α; TGF-β: Transforming growth factor-β; TLR: Toll like receptor.

## Competing interest

The authors declare that they have no competing interest.

## Authors’ contributions

TI designed the research. YR, MH, YS, YY, SY, HT, and MN conducted the experiments. YR, TI and TS analyzed the data and wrote the manuscript. TI and GS had primary responsibility for final content. All authors read and approved the final manuscript.
